# Congenital hyperinsulinism in two siblings with *ABCC8* mutation: same genotype, different phenotypes

**DOI:** 10.20945/2359-3997000000077

**Published:** 2018-10-01

**Authors:** Francisco Sousa-Santos, Helder Simões, Lidia Castro-Feijóo, Paloma Cabanas Rodríguez, Ana Fernández-Marmiesse, Rebeca Saborido Fiaño, Teresa Rego, Ángel Carracedo, Jesús Barreiro Conde

**Affiliations:** 1 Hospital Egas Moniz Lisbon Portugal Serviço de Endocrinologia, Diabetes e Metabolismo, Hospital Egas Moniz, Lisbon, Portugal. Unidad de Endocrinología Pediátrica y Crecimiento. IDIS. Hospital Clínico Universitario de Santiago de Compostela, Santiago de Compostela, Spain; IDIS Unidad de Endocrinología Pediátrica y Crecimiento Santiago de Compostela Spain; Hospital Clínico Universitario de Santiago de Compostela Santiago de Compostela Spain; 2 Instituto Portugues de Oncologia de Lisboa Serviço de Endocrinologia Portugal Serviço de Endocrinologia, Instituto Portugues de Oncologia de Lisboa, Portugal; 3 IDIS Universidad de Santiago de Compostela Hospital Clínico Universitario Santiago de Compostela Spain Unidad de Endocrinología Pediátrica y Crecimiento. Pediatría, Hospital Clínico Universitario y Universidad de Santiago de Compostela, IDIS, Santiago de Compostela, Spain; Unidad de Endocrinología Pediátrica y Crecimiento Santiago de Compostela Spain; 4 IDIS Universidad de Santiago de Compostela Hospital Clínico Universitario Santiago de Compostela Spain Unidad de Endocrinología Pediátrica y Crecimiento. Pediatría Hospital Clínico Universitario y Universidad de Santiago de Compostela, IDIS, Santiago de Compostela, Spain; Unidad de Endocrinología Pediátrica y Crecimiento Santiago de Compostela Spain; 5 Hospital Clínico Universitario de Santiago de Compostela Santiago de Compostela Spain Pediatría, Hospital Clínico Universitario de Santiago de Compostela, Santiago de Compostela, Spain; 6 IDIS Unidad de Endocrinología Pediátrica y Crecimiento Spain Unidad de Endocrinología Pediátrica y Crecimiento, IDIS. Hospital Clínico Universitario de Santiago de Compostela Spain. Endocrinología. Hospital Curry Cabral. Centro Hospitalar de Lisboa Central, Lisboa, Portugal; Hospital Clínico Universitario de Santiago de Compostela Spain Spain; Centro Hospitalar de Lisboa Central Hospital Curry Cabral Lisboa Portugal; 7 Universidad de Santiago de Compostela Universidad de Santiago de Compostela Hospital Clínico Universitario de Santiago de Compostela Fundación Publica Galega de Medicina Xenómica Santiago de Compostela Spain Fundación Publica Galega de Medicina Xenómica, Hospital Clínico Universitario de Santiago de Compostela, Universidad de Santiago de Compostela, Santiago de Compostela, Spain; 8 IDIS Universidad de Santiago de Compostela Hospital Clínico Universitario Santiago de Compostela Spain Unidad de Endocrinología Pediátrica y Crecimiento, Pediatría, Hospital Clínico Universitario y Universidad de Santiago de Compostela, IDIS, Santiago de Compostela, Spain

## Abstract

Congenital hyperinsulinism (CHI) is a heterogenous disease caused by insulin secretion regulatory defects, being *ABCC8/KCNJ11* the most commonly affected genes. Therapeutic options include diazoxide, somatostatin analogues and surgery, which is curative in focal CHI. We report the case of two siblings (born two years apart) that presented themselves with hypoketotic hyperinsulinemic persistent hypoglycemias during neonatal period. The diagnosis of diffuse CHI due to an *ABCC8* compound mutation (c.3576delG and c.742C>T) was concluded. They did not benefit from diazoxide therapy (or pancreatectomy performed in patient number 1) yet responded to somatostatin analogues. Patient number 1 developed various neurological deficits (including epilepsy), however patient number 2 experienced an entirely normal neurodevelopment. We believe this case shows how previous knowledge of the firstborn sibling's disease contributed to a better and timelier medical care in patient number 2, which could potentially explain her better neurological outcome despite their same genotype.

## INTRODUCTION

Congenital hyperinsulinism (CHI) is the most common cause of persistent hypoglycemia in infancy and it can potentially lead to permanent neurological damage (1,2). It is the result of a group of genetic disorders which can lead to severe and persistent hypoglycemia (3). Estimated incidence is 1/50,000 live births (4).

The disease is caused by mutations in various genes related to insulin secretion regulation (5), such as: *ABCC8* (6), *KCNJ11* (7), *GCK* (8), *GLUD1* (9), *HADH* (10), *SLC16A1* (11), *HNF4A* (12), *HNF1A* (13), *UCP2* (14), *HK1* (15) and *PGM1* (16). The most common mutations affect *ABCC8* and *KCNJ11* genes and lead to inactivation of the ATP-dependent potassium channel (K_ATP_) subunits (SUR1 and Kir6.2 respectively), resulting in unregulated insulin β-cell secretion (2,4,7,17).

Histologically the disease is characterized by Langerhans islet hyperplasia and can present as diffuse (18) or focal form (19), which is at least partially determined by the type of genetic defect (3). The two forms are clinically indistinguishable and the best way to differentiate is by performing a 18F-fluoro-L-DOPA positron emission tomography (PET) (20).

Pharmacotherapeutic options (3,21) include oral diazoxide as first line treatment and glucagon, somatostatin analogues and calcium channel blockers. In cases of focal hyperplasia, the treatment of choice is surgical removal of the affected tissue, which is usually curative (19). In diffuse forms that are unresponsive to medical therapy, near-total pancreatectomy can be an alternative, although risking complications such as diabetes (3).

We report the case of two siblings diagnosed in neonatal period with CHI who were found to have the same genetic defect but different longterm disease course. The previous knowledge of the firstborn sibling's disease and how it responds to different therapeutic options led to a more appropriate therapeutic intervention in the second sibling, which we believe contributed decisively to a better neurological outcome, even with the same disease genotype. We believe this is a prime example of how a timely diagnosis and appropriate therapeutic strategy are determinant to a better clinical outcome.

## CASE REPORT - PATIENT 1

An early term large-for-gestational age - birth weight: 4,200 g (SDS +2.84) and APGAR 9/10 - male patient born to nonconsanguineous parents presented with severe and persistent hypoglycemia 36 hours after birth, with impaired consciousness level, hypotonia and bradycardia. This was his 28-year-old mothers first gestation and she developed gestational diabetes at the 20^th^ week (controlled with diet). There was no family history of hypoglycemic disorders. Starting on the fourth day of life, he required continuous infusion of concentration glucose (glucose infusion rate 8-10 mg/ kg/minute) to maintain normal blood glucose.

Abdominal ultrasound and computed tomography did not identify any pancreatic abnormalities. Laboratory studies (Table 1) demonstrated very low levels of ketone bodies and free fatty acids in the presence of low glycemic values and inappropriately high insulin levels. Glucagon stimulation test yielded positive results - blood glucose increased 46.8 mg/ dL 30 minutes after glucagon administration. Genetic analyses, performed at 5 years of age, identified biallelic *ABCC8* mutation - a frameshift mutation, c.3576delG, inherited from his mother and a nonsense mutation, c.742C>T, inherited from an unaffected father.

**Table 1:** Laboratory findings at the time of hypoglycemia

Patient (Age)	Patient 1 (1 month)	Patient 2 (< 1 month)
Blood glucose (70-110 mg/dL)	28	12
Insulin (9.7-97.2 pmol/L)	64.6	110.4
C Peptide (0.30-1.42 nmol/L)	0.9	2.3
Hydroxybutyrate (< 300 μmol/L)	Undetectable	Undetectable
Free fatty acids (0.4-0.7 nmol/L)	0.33	0.43
Cortisol (137.9-689.7 nmol/L)	855.3	634.6
GH (< 20 μg/L)	5.4	11
Carnitines:		
Total (38-68 nmol/mL)	60	42
Free (27-49 nmol/mL)	46	
Acilcarnitines (7-19 nmol/mL)	14	
Lactate (0.6-2.3 mmol/L)	0.9	13.4
Pyruvate (80-160 μmoI/L)	67	54.5
Glutamic Acid (0.2-2.8 mg/dL)	0.77	
Ammonia (10-65 μmol/L)	61	45
Urinary organic acids	Negative	Negative

He was initially started on diazoxide (titrated to 25 mg/kg/day) and later added hydrochlorothiazide. When he was 3 months old, he was submitted to exploratory abdominal surgery and near-total pancreatectomy (> 90%) due to persistent severe hypoglycemic episodes associated with seizures. Histology revealed diffuse pancreatic islets hyperplasia. Postoperatively, there were no improvements, so, at the age of two, he began gastrostomy continuous enteral feeding using complex carbohydrates and was switched to subcutaneous octreotide (20 μg/kg/day).

Neurologic evaluation at 5 years of age demonstrated psychomotor development retardation, hemiparesis, dysesthesias and involuntary movements of the left arm. magnetic ressonance imaging (MRI) showed diffuse cerebral atrophy and a T2 hyperintensity of the medial temporal right lobe, suggestive of hypoglycemic sequelae (22). He was diagnosed with partial epilepsy and started on oxcarbazepine and, subsequently, valproic acid.

**Figure 1:**
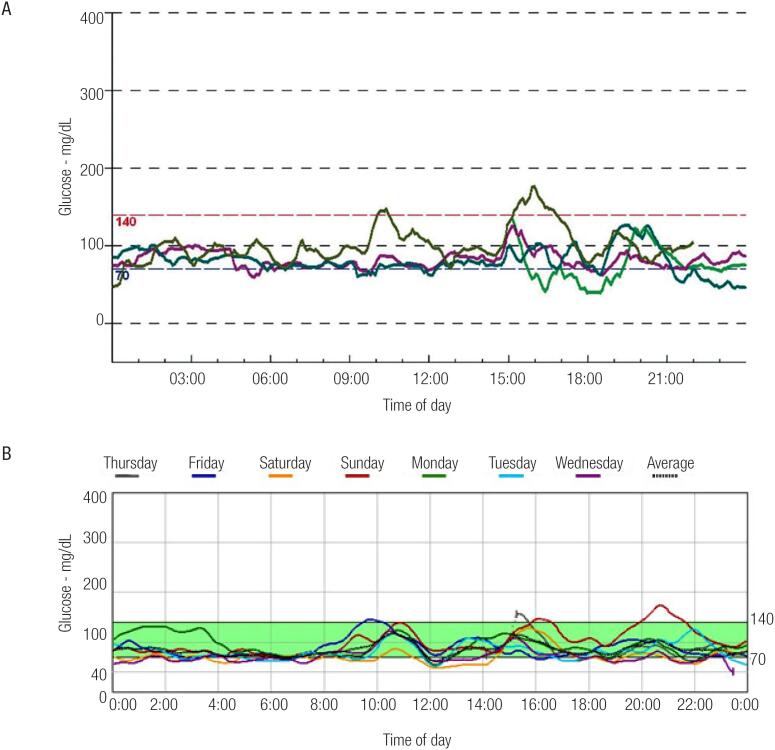
Results of both patients’ subcutaneous glucose monitoring: (**A**) Patient number 1's 24-hour monitoring. Various periods of low blood glucose (40-60 mg/dL) were observed. (**B**) Patient number 2's 6-days monitoring. Various periods of low blood glucose (40-60 mg/dL) were also registered in this case.

The result of a continuous glucose monitoring performed at 13 years of age, while on medical therapy with octreotide and nifedipine, is shown in Figure 1A. By the age of 16 the patient exhibited improvement of his glycemic profile. Octreotide was switched to lanreotide (60 mg each 30 days) when he was 18 years old.

At the time of writing, the patient is 20 years old and remains without any recent hypoglycemic episodes although occasionally postprandially hyperglycemic (diabetes criteria not met). His BMI (body mass index) is 22.8 kg/m^2^, thus showing improvement since infancy (BMI > 97^th^ percentile). He is 169 cm tall (SDS −1.15) (23) and target height was: 177.5 cm (SDS + 0.25). IGF-1 and IGFBP-3 remained normal during all follow-up and pubertal development was adequate. He remains with slight neuropsychological deficits, without recent clinical epileptic activity. He is currently medically managed with lanreotide 60 mg each 30 days, methylphenidate (started at 12 years of age due *\* to attention deficit disorder) and carbamazepine.

## CASE REPORT - PATIENT 2

An early term - birth weight: 3,660 g (SDS +1.64) and APGAR 9/10 - female patient, sister of patient number 1 (born 2 years after him), presented with severe hypoglycemia (20 mgl/dL) one hour after birth. This was her mother's second gestation and she, once more, developed gestational diabetes at the 20th week (managed with dietary measures). During her first days of life she required continuous infusion of glucose (glucose infusion rate 12 mg/kg/minute) to maintain euglycemia.

Abdominal imaging and laboratory studies (Table 1), including glucagon simulation test, revealed similar results as patient number 1. Genetic analyses found the same compound mutation.

At the age of one month, she was started on diazoxide (12 mg/kg/day) and hydrochlorothiazide but showed no benefit. One month later diazoxide was switched to octreotide (20 mg/kg/day) and she was started on continuous gastrostomy enteral feeding. During infancy and adolescence, her hypoglycemic episodes became less frequent and less severe and there were no recorded hyperglycemic episodes - results of continuous glucose monitoring performed when she was 13 are shown in Figure 1B. She was switched to lanreotide (60 mg each 30 days) when she was 16.

Currently the patient is 18 years old and remains with occasional non-severe hypoglycemic episodes but is otherwise euglycemic. Her psychomotor and intellectual development remains normal. BMI values (BMI > 97^th^ percentile) consistent with obesity were observed until she was 6 years old, however her present BMI is 22.6 kg/m^2^. Her final height is 160 cm (SDS −0.60) (23) and target height was: 165.4 cm (SDS+ 0.23). No GH/IGF axis abnormalities were found throughout follow-up. She is currently medically managed with lanreotide 60 mg each 28 days and is on a fractionated diet.

## DISCUSSION

We describe the case of two siblings diagnosed with diazoxide-unresponsive CHI due to the same compound heterozygous *ABCC8* mutation, yet different disease courses.

In the presence of CHI, molecular biology plays an important role in providing a precise diagnosis and potentially guiding the pharmacotherapy (24). *ABCC8* gene mutation are the most commonly found defects in patients with CHI (6). Monoallelic *ABCC8* mutations can cause both diazoxide-responsive and unresponsive CHI, however most biallelic mutations result in diazoxide-unresponsive form (25).

Our patients presented with biallelic *ABCC8* mutations - c.3576delG and c.742C>T (already referred to in a Spanish genetic mutation database (17)). The former mutation, has only been described in heterozygosity and associated with the focal form of the disease (26,27). The latter has been associated with CHI both in homozygosity and heterozygosity presenting as histologically diffuse disease (28,29). Both mutations result in premature termination codons. Therefore, here we describe two cases of a novel (to the best of our knowledge) compound mutation that retains the histological features (diffuse disease based on pancreatic histology of patient number 1) of the c.742C>T mutation. Functional studies could confirm the resultant protein grade of dysfunction, as other authors have done in different compound mutations (25).

The first therapeutic approach usually consists of diazoxide. It inhibits pancreatic insulin secretion through interaction with the SUR1 subunit of the K_ATP_ and therefore in case of abnormalities in these proteins it might not be effective (3,21,30). Such is the case in *ABCC8* mutations, making most of these patients diazoxide-unresponsive, as were these subjects.

In patients with diazoxide-unresponsive CHI, somatostatin analogues, usually administered as subcutaneous injections, have shown some promise (3,21). They act on somatostatin receptors (SSTR2 and SSTR5), inhibiting the secretion of various hormones, namely insulin. They have been used for over 20 years in the long-term control of diazoxide- unresponsive forms of the disease (31), however they have yet to be officially approved for this purpose. In addition to its side-effect profile (which includes gastrointestinal symptoms, biliary lithiasis and rare cases of necrotizing enterocolitis), there is a risk of worsening hypoglycemia (due to suppression of GH and glucagon production) and tachyphylaxis (due to downregulation of somatostatin receptors), which can limit its long-term use (3,21). Octreotide is the drug with the most clinical experience, however in the last years there have been reports of successful lanreotide (long-acting somatostatin analogue) use (32). The presented cases represent how both octreotide and lanreotide can be effective in attaining blood glucose control both post (patient number 1) and pre-operatively (patient number 2) in case of diffuse diazoxide-unresponsive CHI. These drugs seemed better tolerated than diazoxide.

Alternative pharmacotherapy options include nifedipine (a calcium channel blocker that has had mixed results (18)) and glucagon (used for short-term control of hypoglycemias (31)). Dietary treatment (21) can be sufficient in some cases and involves frequent glucose enriched oral feedings and continuous or frequent enteral feedings.

Surgical removal remains the best option in cases of an identifiable focal lesion associated with CHI – 94% can be rendered euglycemic at the time of discharge (33). In cases of medically refractory diffuse CHI, near-total pancreatectomy (95-98%) may be the only therapeutic option to cure or at least facilitate the medical management. The surgical outcome in case of diffuse CHI is highly variable: persistent hypoglycemia in about 50% of patients and diabetes in 20%, however most improve their glycemic control (3). This option must be carefully weighted also due to its potential complications (namely diabetes and exocrine pancreatic insufficiency (34)) and the fact that spontaneous remission can be observed in some cases (31). Regarding our cases, near-total pancreatectomy was performed in patient number 1 due to the severity and neurological repercussions of his hypoglycemic events and the lack of alternative pharmacotherapeutic options, even though by that time there was not a molecular diagnosis nor any other data to estimate the pancreatic extent of the disease (PET was not available and there were no macroscopic pancreatic lesions on surgical exploration). There was no clinical benefit post-operatively and this contributed to the decision to withhold surgery in patient number 2 - also given the new-found knowledge of the extent of CHI (diffuse involvement based on patient number 1 pancreatic histology) associated with this mutation.

There have been reports of patients with CHI due to HNF4A (35) and HNF1A mutations (36) who present initially with hyperinsulinemic hypoglycemia and develop maturity-onset diabetes of the young type (MODY) later in life. ABCC8 mutations have been implied in cases of neonatal diabetes (frequently transient), MODY (37) and there are reports of hyperinsulinemic hypoglycemic disorder in early life progressing to insulinopenic diabetes later on (38). Regarding our cases, patient number 1 continues to record periods of post prandial hyperglycemia, although diabetes has been excluded using oral glucose tolerance test and glycated hemoglobin measurement. This phenomenon is most likely a complication of neartotal pancreatectomy rather than another feature of her disease/genotype. Additional support to this hypothesis is presented by the fact that there were no hyperglycemic episodes recorded to date in the case of patient number 2, with the same disease genotype. Nevertheless, given these cases concern a new compound mutation, the risk of progression to diabetes in adulthood is largely unknown and should not be disregarded.

An interesting finding is the fact that the mother developed gestational diabetes in both pregnancies, although hyperglycemia did not persist after deliveries. It has already been established that maternal hyperglycemia can induce fetal hyperinsulinemia and be responsible for macrosomia and neonatal hypoglycemia (39). Nevertheless, no link has ever been implied between maternal hyperglycemia and the presence of insulin regulatory gene mutations (the mother was found to be carrier of the *ABCC8* c3576delG mutation).

Prolonged and severe hypoglycemias expose patients to a poor neurological outcome and, indeed, some series report neurodevelopment delay issues in as much as 30% of CHI patients (18). This risk may be higher in cases of neonatal onset and diazoxide-unresponsive CHI (3). Some MRI brain injury patterns have been identified (22), such as cerebral cortex T2 hyperintense lesions while sparing cerebellum, brainstem, and thalamus - this was the case of patient number 1. Taking this into account, the better neurodevelopment observed in patient number 2 can probably be linked to a more rapid and appropriate treatment approach due to the previous knowledge of her sibling's disease. In particular, octreotide was initiated when she was 2 months old while her brother only started at 2 years of age, which probably led to a lesser frequency and severity of hypoglycemic episodes during her infancy and could explain her better neurological outcome.

In conclusion, we report the case of two siblings with diazoxide-resistant CHI caused by the same compound *ABCC8* mutation. Their therapeutic management had some differences and this could offer a potential explanation for the distinct neurological outcome, despite the same genetic basis for the disease. This case highlights the importance of the first siblings index case clinical and molecular diagnosis and how it seemed to contribute to a better disease knowledge and medical care in patient number 2, ultimately resulting in a better outcome in spite of the same disease genotype.
